# Favipiravir Experience in Covid-19 Positive Myasthenia Gravis Patients

**DOI:** 10.5152/eurasianjmed.2021.20364

**Published:** 2021-06

**Authors:** Recep Yevgi, Nuray Bilge, Fatma Şimşek

**Affiliations:** Department of Neurology, Ataturk University School of Medicine, Erzurum, Turkey

Dear Editor:

Myasthenia gravis (MG) is a disease that occurs as a result of an immunological attack against various proteins in the postsynaptic membrane, especially the postsynaptic acetylcholine receptors at the neuromuscular junction. Coronavirus disease (COVID-19), caused by the new virus severe acute respiratory syndrome coronavirus 2, became a worldwide pandemic. Although there is no definitive treatment for the disease as of yet, agents such as chloroquine, hydroxychloroquine, lopinavir, ritonavir, favipiravir, and remdesivir are used in many countries based on in vitro or observational studies. Considering that certain drugs can cause myasthenic exacerbation in patients with MG, the consequences of using drugs such as favipiravir in these patients are unknown. We aim to share our experience using favipiravir in 2 patients previously diagnosed with MG who tested positive for COVID-19.

A 32-year-old female patient with MG presented to our clinic with complaints of fever, cough, shortness of breath, difficulty swallowing, and fatigue for the last 2 days. The patient’s body temperature was 37.9 °C, C-reactive protein (CRP) level was 125 mg/dL (normal: 0–5 mg/dL), white blood cells (WBC) were 12,640/μL (normal: 5500–11,000/μL), and lymphopenia was present (0.81 x 10^3^/μL). Thoracic computed tomography (CT) revealed patches of ground-glass opacities and consolidation in the lung parenchyma consistent with COVID-19 pneumonia. The patient’s COVID-19 real time-PCR test was positive. IVIg was initiated for myasthenic crisis, and favipiravir was initiated for COVID-19. During follow-up, the patient’s complaints of shortness of breath and difficulty swallowing improved. Follow-up thorax CT was taken 15 days later, and it was observed that ground-glass appearances were reduced. The patient was found to be stable at follow-up and she was discharged.

A 56-year-old male patient with MG presented with complaints of shortness of breath, difficulty swallowing, high fever, and muscle pain which began 1 day ago. The patient’s body temperature was 38.8 °C, CRP level was 224 mg/dL (normal: 0–5 mg/dL), WBC was 19,000/μL (normal: 5500–11,000/μL), and lymphopenia was present (0.43 x 103/μL). Arterial blood gas was as follows: pH: 7.23, PCO_2:_ 94.9 mmHg (normal: 32 mmHg–45 mmHg), PO_2_: 84.5 mmHg (N: 75 mmHg–100 mmHg), and HCO_3_: 38.7 mmol/L (normal: 21.2 mmol/L–28.3 mmol/L). The patient was intubated owing to respiratory acidosis and dyspnea. Thorax CT revealed focal ground-glass densities and tree-in-bud patterns in the right upper lobe anterior segment of the right lung. COVID-19 RT-PCR test was positive. IVIg was administered for myasthenic crisis and favipiravir was administered for COVID-19. As the patient’s respiratory acidosis in blood gas resolved on day 12, the patient first received continuous positive airway pressure (CPAP) then nasal cannula. General supportive care continued. The patient was found to be stable at follow-up and his shortness of breath and difficulty swallowing resolved. The patient was discharged.

Although there are limited studies on COVID-19 progression in patients with MG, the use of immunosuppressive and immunomodulatory therapies in many of these patients causes concern among clinicians. There are many studies that report that hydroxychloroquine and azithromycin, which are widely used to treat COVID-19, may lead to myasthenic exacerbation.^1^–^3^ Therefore, we chose to use favipiravir rather than hydroxychloroquine and azithromycin in both of our patients. In our first case, no side effects or myasthenic exacerbation associated with favipiravir were observed. In our second case, we did not observe any adverse effects due to favipiravir, but our experience regarding myasthenic exacerbation was limited owing to the patient being intubated for some time.[Table t1-eajm-53-2-164]

## Figures and Tables

**Table 1 t1-eajm-53-2-164:** Patient Characteristics

	Case 1	Case 2
Age (sex)	32 (F)	56 (M)
Duration of diagnosis, years	3	2
Comorbidities	No	Hypertension
Blood or urine cultures	No growth	No growth
Antibody status	AChR+	Seronegative
Thymic pathology	Yes	No
Thymectomy (pathology)	Yes (TypeB3)	No
Neurologic examination	Bilateral orbicularis oculi were weak, and MRC scores as neck flexor muscles 3/5, bilateral lower and upper extremities as 4/5 muscle strength.	Bilateral orbicularis oculi were weak, and MRC scores as neck flexor muscles 2/5, bilateral lower and upper extremities as 4/5 muscle strength
MGFA score at the time of COVID-19	IIB	V
MGFA score prior to infection	I	I
MG treatment	Prednisolone 25 mg/day Pyridostigmine 60 mg 4×1	Prednisolone 35 mg/day Pyridostigmine 60 mg 3×1
MG treatment change during COVID 19 infection	No change	Pyridostigmine stopped during intubation period
Treatment for myasthenic exacerbation	IVIG 0.4gr/kg/day (5 days)	IVIG 0.4gr/kg/day (5 days)
Treatment for COVID-19	Favipiravir 2×1600 mg loading dose then 2×600mg maintenance dose (5 days)	Favipiravir 2×1600 mg loading dose then 2×600mg maintenance dose (5 days)
Adverse effects related to favipiravir	No	No
Myasthenic exacerbation related to favipiravir	No	Limited due to intubation
